# Symptomatic calcified chronic subdural hematoma in an elderly patient: a case report

**DOI:** 10.1186/s13256-023-04078-7

**Published:** 2023-08-15

**Authors:** Esayas Adefirs Tefera, Mezgebu Alemneh Assefa, Yohannis Derbew Molla

**Affiliations:** Department of Surgery, Collage of Medicine and Health Sciences, University of Gondar, Gondar, Ethiopia

**Keywords:** Calcified hematoma, Subdural, Headache, Case report

## Abstract

**Introduction:**

Calcified chronic subdural hematoma is a rare and infrequent diagnosis made in clinical practice according to the literature. Calcification of chronic subdural hematoma is found more frequently in children and young adults than in the aged. The proposed mechanism of calcification may involve poor circulation and absorption in the subdural space together with intravascular thrombosis and prolonged existence of the hematoma in the subdural space.

**Clinical presentation:**

An 84-year-old Ethiopian male patient presented with progressive right-sided body weakness of 8-month duration. The weakness started in the right lower extremity and progressively involved the upper extremity. Associated with the above complaint, he had had also a globalized headache of the same duration. Pre- and post-contrast brain computed tomography scans showed a right hemispheric extra-axial collection that crossed the suture line, with a maximum depth of 2.3 cm. Subsequently, craniotomy and hematoma evacuation were carried out and the patient was discharged improved.

**Conclusion:**

The most common symptom of calcified chronic subdural hematoma is headache followed by lethargy, confusion, memory impairment weakness, and seizures. A diminished level of consciousness is relatively common and motor deficits are usually manifested as hemiparesis or gait disturbance. Most calcified chronic subdural hematomas can be diagnosed by computed tomography or magnetic resonance imaging and differentiated from the usual chronic subdural hematoma by imaging studies and gross pathology. Surgical treatment is advised in symptomatic patients when feasible, and often results in neurological improvement. Here we presented a patient with an uncommon calcified chronic subdural hematoma, which was successfully evacuated, resulting in a good recovery.

## Introduction

Calcified chronic subdural hematoma is a rare and infrequent diagnosis made in clinical practice according to the literature. Calcified or ossified subdural mass covering the most of cortical surface is also known as an armored brain. The first case was reported in 1884 [1]. Although chronic subdural hematoma (CSDH) is a well-known disease entity, calcified chronic subdural hematoma (CCSDH) is not common. The incidence of CCSDH has been reported to be 0.3–2.7% of all CSDHs [2]. Calcification of chronic subdural hematoma is found more frequently in children and young adults than in the aged [3]. The pathogenesis of calcification of CSDH is not well defined. Vascular, metabolic, and/or local factors could play a role in the development of this disease process. The most common symptom of CCSDH is headache followed by lethargy, confusion, memory impairment, weakness, and seizures. Altered mentation is relatively common and motor deficits are usually manifested as hemiparesis, such as in our patient, or gait disturbance [4]. Surgery for calcified chronic subdural hematoma is generally recommended for infants and young patients, or patients with progressive neurological deficits, increased intracranial pressure, or intracerebral hematoma [5]. Here we present a case of CCSDH in a geriatric patient who was subsequently managed surgically and discharged improved.

## Clinical presentation

An 84-year-old Ethiopian male patient presented with progressive right-sided body weakness of 8-month duration. The weakness started in the right lower extremity and progressively involved the right upper extremity. The weakness was initially mild and he was able to move with support, but later it became worse and he was unable to walk even with assistance. Associated with the above complaint, he had had a globalized headache of the same duration. However, he had no history of loss of consciousness, abnormal body movement (seizure), early morning vomiting, confusion, swallowing difficulty, or lethargy. He had no history of personality change, memory loss, or language deficit. He had no history of impaired vision or double vision. He had no history of trauma to the head. He had no history of bleeding tendencies or coagulopathies. He had no history of diabetes mellitus, hypertension, asthma, cardiac illnesses, or history of drug intake. He has no history of any known allergies. He has no history of chronic alcohol ingestion. He has no history of surgery or any other intervention.

**Fig. 1:**
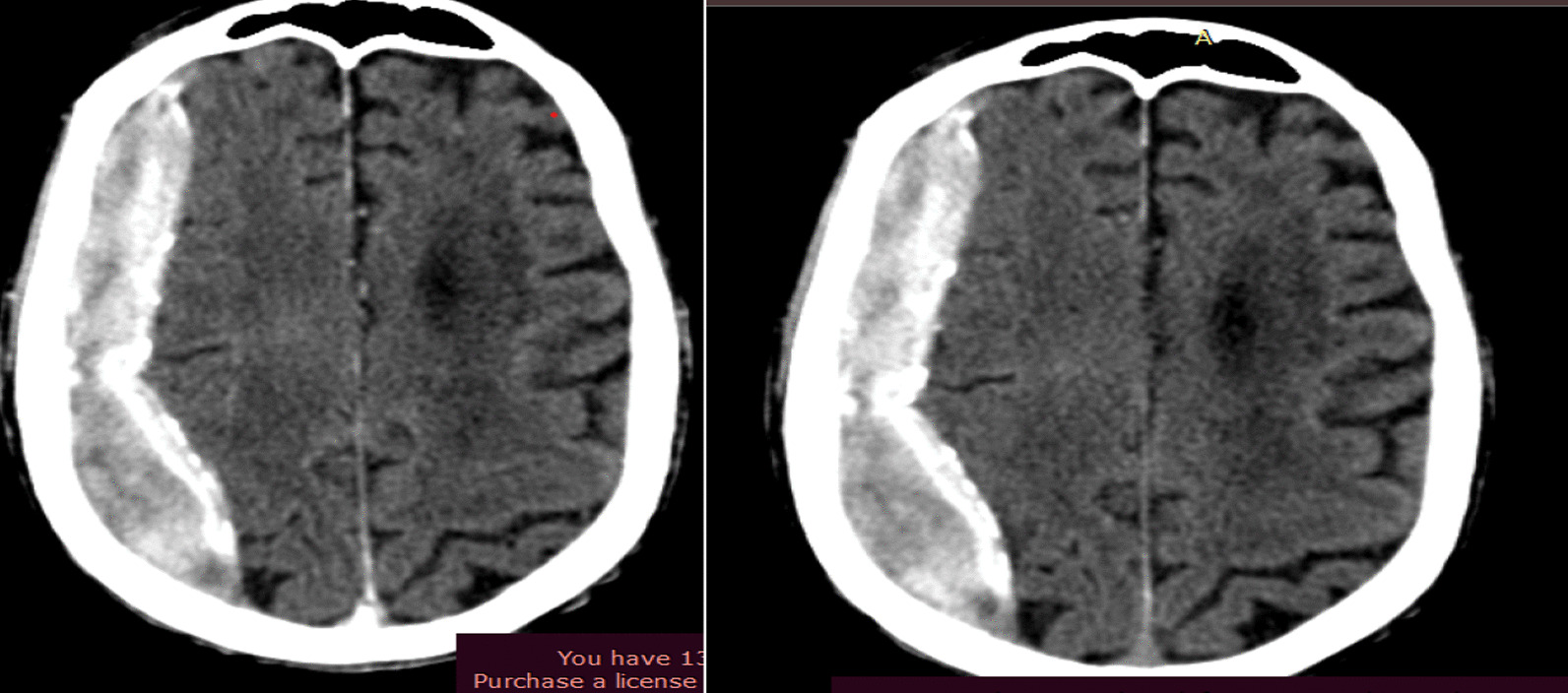
Pre- and post-contrast computerized tomography scan showing calcified subdural hematoma

On examination at admission, his vital signs were within the normal range. He had a clear and resonant chest. On central nervous system examination, his Glasgow coma scale (GCS) was 15/15 and his pupils midsize and reactive bilaterally. On motor examination, the right upper extremity was 4/5 and hypertonic, the right lower extremity was 3/5 and hypertonic, and left upper and lower extremities were 5/5 and normotonic. The sensation was intact all over the body. Deep tendon reflexes were normal on both upper and lower extremities. The rest of his examinations were unremarkable. On investigation, his complete blood count and organ function tests were within the normal range. Pre- and post-contrast brain computed tomography (CT) scans showed a right hemispheric extra-axial collection that crossed the suture line with a maximum depth of 2.3 cm with effacement of ipsilateral lateral ventricle and midline shift of 3 mm. The collection was heterogeneously hyperdense with diffuse thick calcification with no post-contrast enhancement. The rest of the cortical sulci and ventricles were prominent. Otherwise, no abnormal brain parenchymal, meningeal, or vascular enhancement was seen (Fig. 1).

**Fig. 2:**
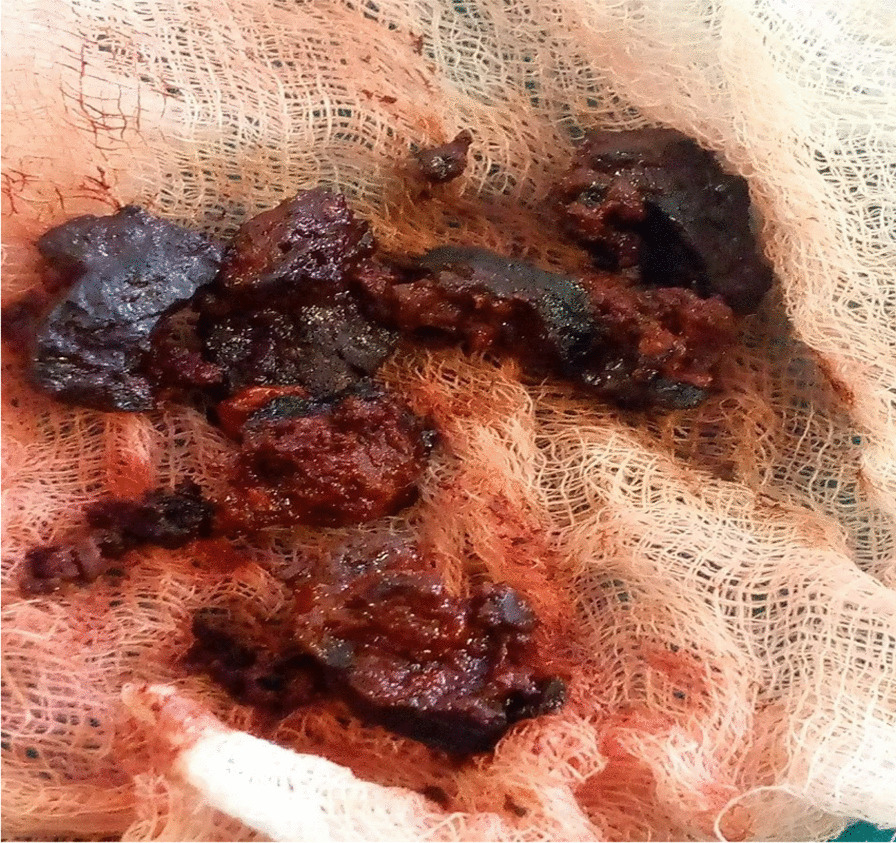
Gross appearance of the calcified hematoma after surgical removal

Subsequently, the patient was resuscitated and diagnosed with right CCSDH, and right parietal craniotomy, hematoma evacuation, and excision was carried out. The patient was operated on under general anesthesia and supine position by a neurosurgeon and general surgery residents. The duration of the surgery was 1.5 hours. Intraoperatively, the dura was calcified, and there was a hard and calcified hematoma in the subdural space, with the brain pushed to the contralateral side. Therefore, the calcified hematoma was removed gently with a brain dissector as much as possible (Fig. 2), the calcified hematoma firmly attached to the brain was left behind as it was difficult to remove without damaging the brain. With hemostasis secured, a drain was left in the subdural space, the bone replaced back, and the skin closed in layers. Later the patient was put on prophylactic antibiotics (ceftriaxone 1 g intravenous twice a day), phenytoin 100 mg orally three times a day, analgesics (paracetamol 1 g orally three times a day), physiotherapy, and wound care. On subsequent follow-up at the recovery room and in the wards, the patient was followed with neuro-sign chart, the patient had minimal drain output, weakness improved significantly, and headache decreased as well. Finally, the patient was discharged improved with no postoperative complications after 4 days of hospital stay. On follow-up at the surgical referral clinic at 2 weeks, 3 months, and 6 months, the above symptoms did not recur and he was able to walk unsupported.

## Discussion

Subdural hematoma is a collection of blood between the dura mater and the arachnoid, covering one or both cerebral hemispheres, as a result of the tearing of the bridging veins in the region of the sagittal sinus [6]**.** Chronic subdural hematoma (CSDH) is defined as the “collection of blood on the brain’s surface, under the outer covering of the brain presenting more than 21 days.” CSDH usually occurs after injury. However, the trauma may be trivial and the patient may not remember. For example, our patient had no known trauma history or was not able to remember [1]. Calcified chronic subdural hematoma (CCSDH) was first reported by von Rokitansky during an autopsy. Calcification usually develops at an interval longer than 6 months, especially in children and young adults, although it can happen in elderly patients as well. The duration of the symptoms in our patient was 8 months. Whether the development of CCSDH follows a regressive or progressive course is still controversial [2]. The process of calcification of chronic subdural hematoma can result from “regressive” changes, such as poor absorption in the subdural space, calcium deposition, and hyalinization of connective tissue, rather than from an “active” process, but the exact mechanism of this process remains to be defined [7]. Certain predisposing factors, such as alcoholism and coagulopathy, have a role in the development of these lesions, and neurodegenerative diseases including dementia in elder patients should be considered in the differential diagnosis of the hematoma. However, our patient did not have any known risk factors [7]. In some of the reported cases, there has been an antecedent history of cranial injury or illness in childhood of many years preceding the discovery of the skull findings. This has led to the theory that the radiological findings in the examination of the skull are the result of a progressive series of physiological and anatomical changes. In children, trauma can frequently go undetected, especially at a very young age [8]. The CCSDH has been associated with brain atrophy, thus a hematoma may not cause a mass effect. In addition, a calcified hematoma may sometimes tightly adhere to the dura mater and cortex and dissection from the brain may cause brain contusion or bleeding. Therefore, the removal of this lesion had not been considered necessary or beneficial. However, a CCSDH can be an active lesion that grows like a neoplasm. There is also risk of hemorrhage, as evidence of vascular proliferation has been noted in the capsule of calcified chronic subdural hematoma. Thus, surgical intervention is favored for progressively enlarging lesions [9].

Although the pathogenesis of calcification of CSDHs is still not completely known, local, metabolic, and vascular factors are assumed to play the most important role in the development of calcification and ossification. Calcification occurs between 3 months and 12 months after onset. Calcification of a CSDH is more frequent than its ossification, as ossification may be considered a terminal phase of the process [10]. Recently, it has been reported that some inflammatory markers (interleukins 6 and 10, chemokines, etc.) were elevated in cases with CSDH and enhanced expressions of angiogenic growth factors, vascular endothelial growth factor, and basic fibroblast growth factor in the outer membrane were associated with recurrence of CSDH [6]. The extent of the calcification in CCSDH varies widely, involving the convexity and sometimes the entire hemispheric surface. The CCSDH in our patient was at the convexity [2]. The course of the development of calcification may progress gradually from hyalinization to calcification, and finally ossification through irritation of the tissue. After hemorrhage, calcification usually takes 6 months to many years to develop. The proposed mechanism of calcification is that poor circulation and absorption in the subdural space, together with intravascular thrombosis, prolonged existence of the hematoma in the subdural space, stagnant blood due to insufficient arterial supply and inadequate venous return, thick connective tissue membrane, and other local factors, are considered to contribute to the development of calcification of the chronic subdural hematoma [11]. The CCSDH has been associated with brain atrophy, thus a hematoma may not cause a mass effect. In addition, a calcified hematoma may sometimes tightly adhere to the dura mater and cortex and dissection from the brain may cause brain contusion or bleeding [2].

**Fig. 3:**
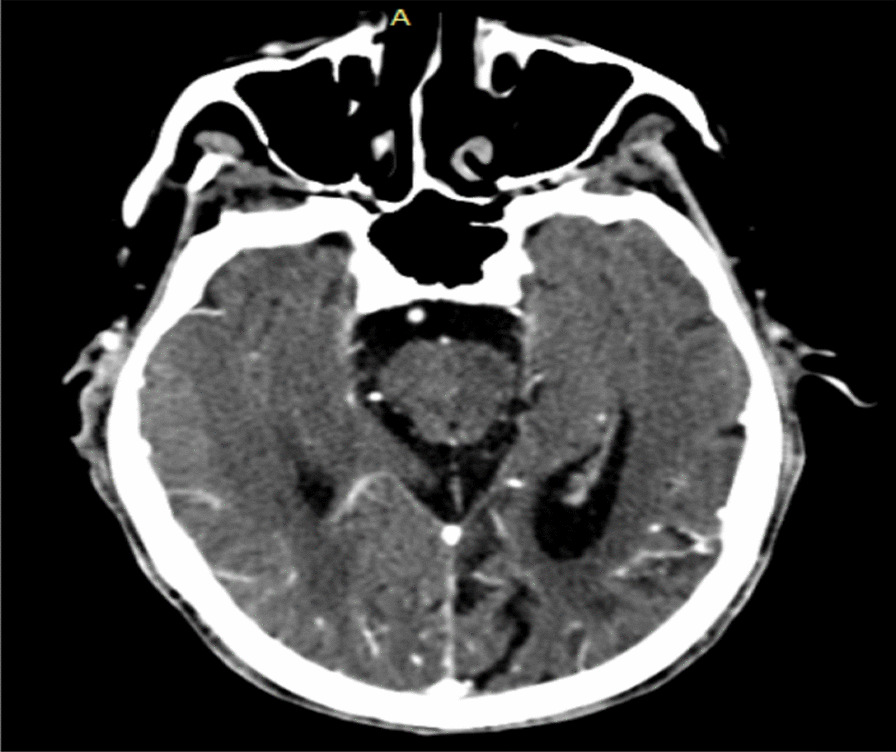
Axial computerized tomography at the level of tentorial hiatus

The most common symptom is headache followed by lethargy, confusion, memory impairment weakness, and seizures. Altered mentation is relatively common and motor deficits are usually manifested as hemiparesis or gait disturbance [4]. Headache and hemiparesis were the presenting symptoms in our patient. The usual expected hemiparesis is contralateral to the hematoma, while our patient had ipsilateral weakness. Such finding is mainly seen in acute hematoma due to Kernohan’s phenomenon, or in a very rare population, without pyramidal decussation. We believe our patient’s finding is due to the latter as there was no contralateral impingement of the midbrain on the tentorial hiatus (Fig. 3) [12]. Most CCSDHs can be diagnosed by CT or magnetic resonance imaging (MRI) and are differentiated from the usual CSDH by imaging studies, and gross pathology CCSDHs have the following characteristics: elliptical shape with the longest diameter in the frontotemporal direction, as shown in Fig. 3; biconvex or flat shape on cross-section; gelatinous or clay-like content, but not liquefied; and thick inner membrane with sinusoidal blood vessels, the inner membrane is partially adhered to and evaginated into the cerebral cortex. Our patient was diagnosed with CCSDH because the CT showed a chronic subdural hematoma with a calcified wall and the CCSDH had all the characteristics previously mentioned, including the inner membrane adhered to the brain. The differential diagnoses of CCSDH may include other calcified extra-axial space-occupying lesions, such as calcified epidural hematoma, calcified subdural empyema, meningioma, calcified arachnoid cyst, and calcified convexity dura mater with acute epidural hematoma. Among these diseases, CCSDH is most often confused with calcified subdural empyema, and intraoperative aspiration of pus is often needed to confirm the diagnosis [2]. Our patient had complete intradural mass, which rules out calcified epidural hematoma. We did not consider calcified arachnoid cyst as we did not encounter cerebrospinal or clear fluid in the lesion. Though pathologic confirmation is lacking, we did not encounter any tissue to consider meningioma.

Radiological investigations were initially limited to skull x-rays prior to the CT scan era. With the availability of the CT scan, diagnosis has now become easy, and various types of CT scan appearances have been described. Debois and Lombaert were the first to report a calcified chronic subdural hematoma on a CT scan in 1980. A typical picture has been reported, termed armored brains, when the entire hemisphere is involved [13].

Conservative treatment is usually indicated in elderly patients with no neurological symptoms. In younger patients, or when a neurologic deficit is present, such as in our patient, surgical treatment is widely accepted. Removal of subdural calcified mass improves cerebral blood flow, reduces cerebral irritation, and usually leads to neurological improvement. Most studies have shown that complete surgical removal of calcified subdural hematoma with craniotomy is beneficial for symptomatic patients with clinical deterioration [1]. However, even in asymptomatic calcified chronic subdural hematoma, surgical removal should be considered in patients with significant cerebral compression shown on CT scans that are not too advanced in age to prevent additional brain atrophy. Once neurological deficits due to brain atrophy appear, it is difficult to obtain a satisfactory improvement despite surgical removal [5]. Even a thin, calcified hematoma can sometimes tightly adhere to the dura mater and cortex. Dissection from the brain may cause brain contusion or bleeding. Both the outer and inner capsules and the hematoma itself were thickly calcified in our case, and the thickness of the calcified layer was 5 mm. Piecemeal removal of components was possible, with areas of adherent dura removed together with the piece. Gelatinous dark matter was encountered in between the inner and outer membranes, and it was easily removed with suctioning. The most adherent inner membrane was left back and cauterized for hemostasis and possible disruption of angiogenesis. Although we do not have access to one, a high-speed air drill used to shave away the calcification appeared as a possible method for removing the thickly calcified layer without causing brain damage. Through this technique, the calcified hematoma could be decreased to a thin, breakable layer [14]. During the operation, precautions should be taken to prevent the formation of a new subdural hematoma that could have arisen from an injury to the bridging veins situated between the ossified hematomas and the cerebral cortex. If necessary, the microsurgical technique can be used [7].

One of the most frequent complications that may be observed after chronic subdural hematoma operations is recurrent hemorrhage. The primary reason in recurrent hemorrhage is believed to be inadequate brain expansion following hematoma drainage, which develops after prolonged compression. However, since calcified or ossified subdural hemorrhages are rather rare, there is insufficient information regarding the recurrence rate in the literature [15]. Finally, our patient claimed he was satisfied with the care.

A limitation of the case report is that histopathology of the excised sample should have been carried out to confirm the diagnosis and rule out differential diagnoses.

## Conclusion

We described a case of an elderly patient with a rare calcified chronic subdural hematoma who also had rare ipsilateral weakness. The calcified components were successfully removed with a craniotomy and careful piecemeal removal, and the patient made a full recovery. In light of our patient’s experience, we advise surgical therapy for CCSDH in symptomatic patients whenever possible because it frequently leads to neurological improvement. However, during the procedure care should be taken to avoid creating a new subdural hematoma that might result from harm done to the bridging veins.

## Data Availability

The authors of this manuscript are willing to provide any additional information regarding the case report.

## Abbreviations

CCSDH: Calcified chronic subdural hematoma
CT: Computerized tomography
MRI: Magnetic resonance imaging
